# Data based investigation of the energy metering type, billing and usage of sampled residents of Ota Community in Nigeria

**DOI:** 10.1016/j.dib.2018.07.047

**Published:** 2018-07-27

**Authors:** Aderibigbe Israel Adekitan, Bukola B. Adetokun, Alex Aligbe, Tobi Shomefun, Abidemi Orimogunje

**Affiliations:** a Department of Electrical and Information Engineering, Covenant University, Ota, Nigeria; b Department of Electrical Engineering, Pan African University Institute for Basic Sciences, Technology and Innovation (PAUISTI), Jomo Kenyatta University of Agriculture and Technology (JKUAT), Kenya

**Keywords:** Energy metering and billing, Equipment profile, Residential consumers, Energy usage, Alternative energy sources

## Abstract

Accurate energy metering and billing is a challenge in some developing countries. In Nigeria, the issues of inadequate power generation, transmission and distribution infrastructure are recurrent problems, coupled with inefficient energy metering which is a major problem that results in residential energy consumers being billed unfair energy charges by the Electricity Distribution Companies (DISCOs) for unused energy, and this has been termed “crazy bill”. For the energy sector to be effective, energy bills should be based on the actual energy usage and likewise customers must pay for used energy. To achieve this, the Nigerian Electricity Regulatory Commission (NERC) recommended the installation of prepaid meters for all customers, but as at today, there is no full compliance with this regulation. Power supply is grossly unreliable, and this has affected power quality due to frequent load shedding and power outages. The dataset presented in this article captures the type of apartment, the type of electrical appliances used by occupant, the average monthly energy bill paid for electricity, the use or non-use of alternative energy sources, the type of alternative energy sources used, and the type of energy meter used by sampled residents of the Ota community in Ogun State, Nigeria. The dataset was acquired using an investigative questionnaire to survey the residential consumers within the sampled space.

**Specifications Table**

**Table:** 

Subject area	*Electrical Engineering*
More specific subject area	*Energy Metering and Billing, Electrical Equipment Profiling*
Type of data	*Tables, figures and spread sheet file*
How data was acquired	*Data acquisition using an investigative questionnaire, structured with targeted questions to determine respondent׳s energy consumption and the mode of energy metering and billing*
Data format	*Raw, filtered, analysed*
Experimental factors	*Residents of estates where energy dependence is solely on off-grid, estate-owned power generation schemes were excluded from the study. Only residents that use the public power supply from the Power Distribution Company were sampled*
Experimental features	*Frequency distributions and statistical analysis were performed to illustrate metering and billing practices, available electrical energy sources, average DISCO power supply duration and the consumers’ electrical equipment profile*
Data source location	*Residents of Ota community, around Covenant University in Nigeria*
Data accessibility	*The dataset is available in a spreadsheet file attached to this article*

**Value of the data**

• The dataset presents the energy metering, billing and usage of a sampled community in Nigeria which is representative of the general consumer experience in Nigeria, and as such, the dataset may serve as an indicator for the level of supply of prepaid meters by Electricity Distribution Companies (DISCOs) to residential consumers, in line with the directives of the Nigerian Electricity Regulatory Commission (NERC).
• The dataset may be of interest to researchers studying the billing experience of consumers that are still using analog meter for electricity metering.
• The tables, frequency distribution, and figures presented can provide vital insights which may enable these data to be compared with similar data collected in other geographical locations within the country for billing and metering pattern recognition based on consumer׳s location.
• These data may be useful for future studies comparing different methodological approaches to consumer billing and tariff rate determination.
• The availability of this data may trigger similar evidenced based empirical research studies [1], and this may create platforms for extensive collaboration.

## 1 Data

**Fig. 1:**
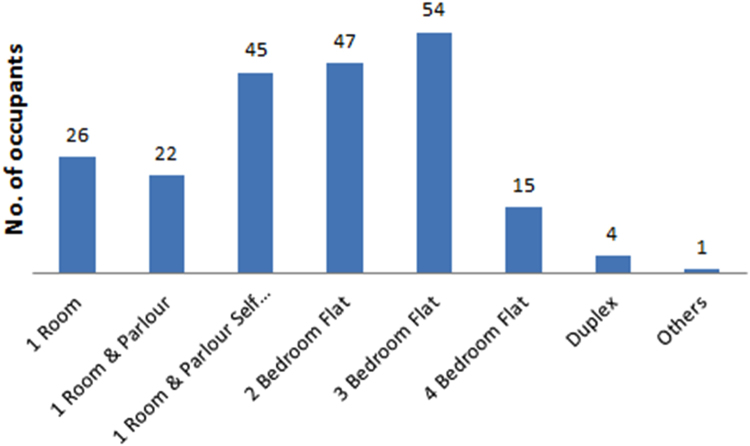
The types of apartment surveyed.

**Fig. 2:**
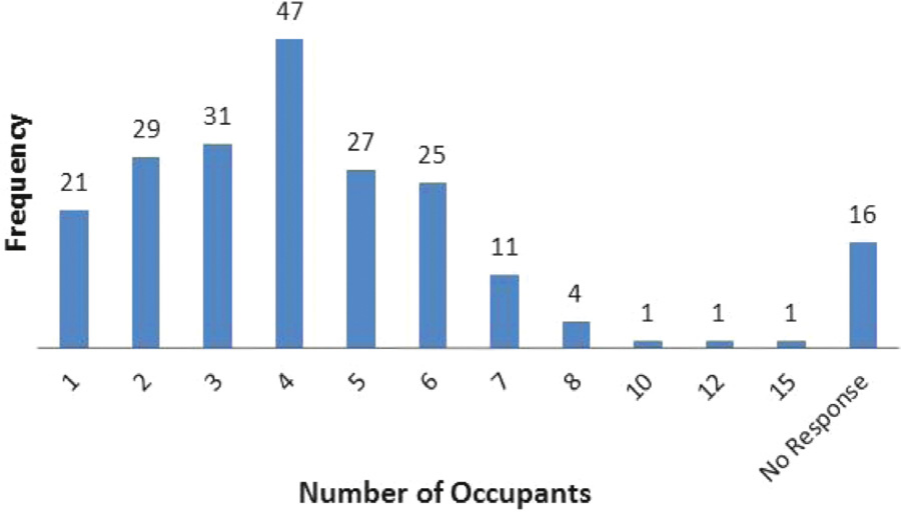
The number of occupants per household.

**Fig. 3:**
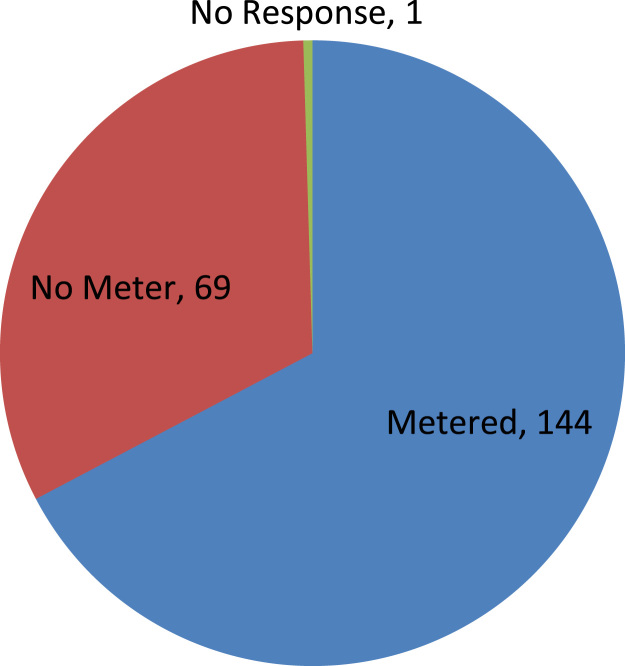
Energy meter availability in households.

**Fig. 4:**
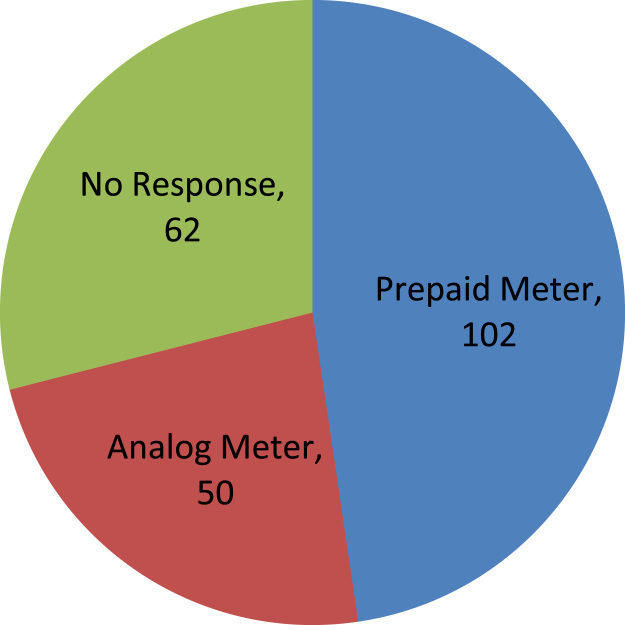
The type of energy meter installed.

**Fig. 5:**
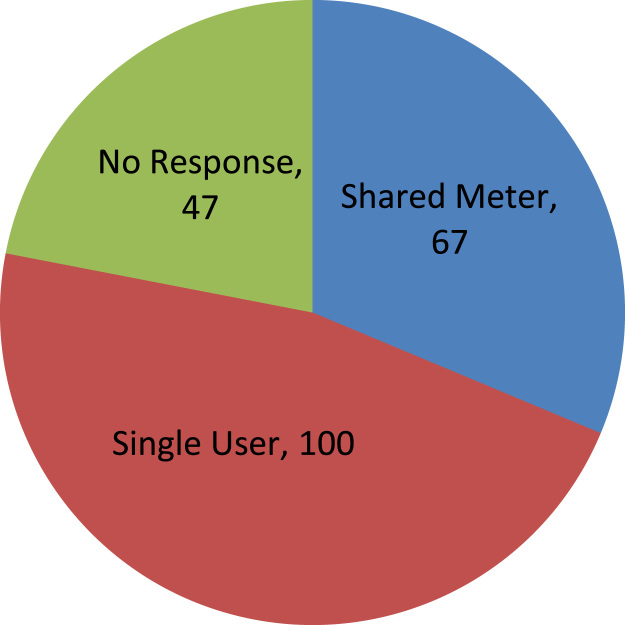
Sharing of one energy meter with landlord or neighbor.

**Fig. 6:**
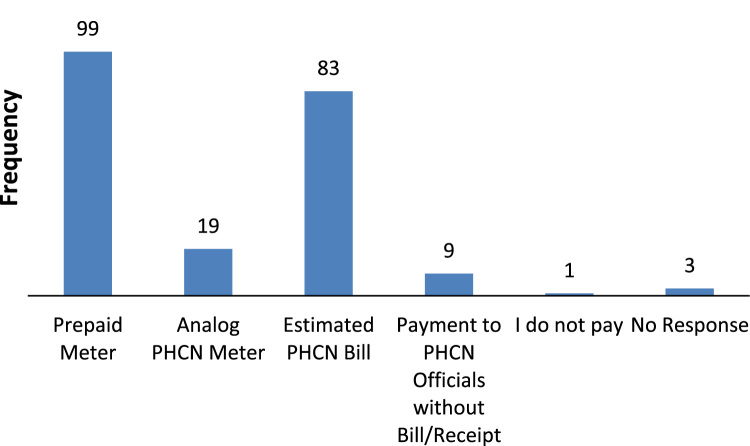
Mode of energy billing and payment.

**Fig. 7:**
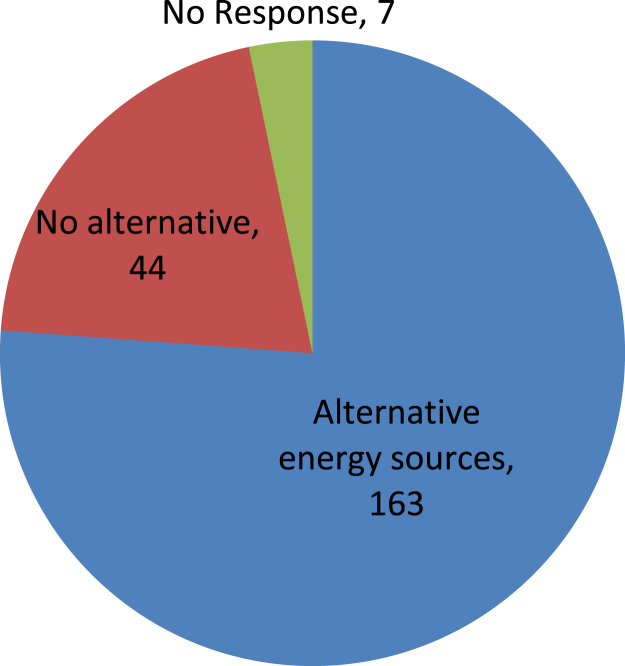
The use of alternative energy sources by Respondents.

**Fig. 8:**
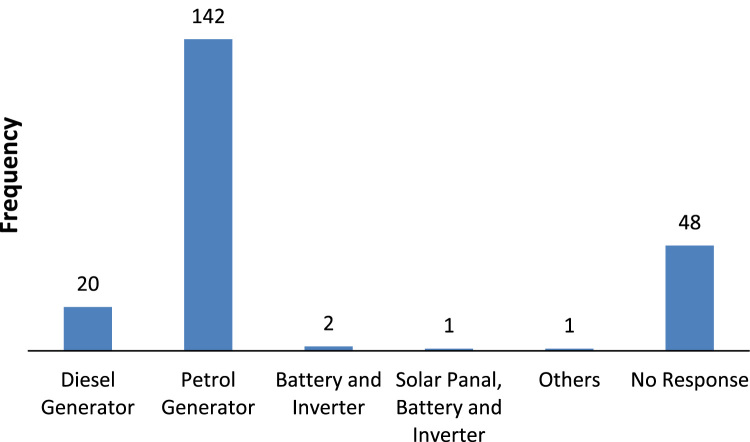
The type of alternative energy source used.

**Fig. 9:**
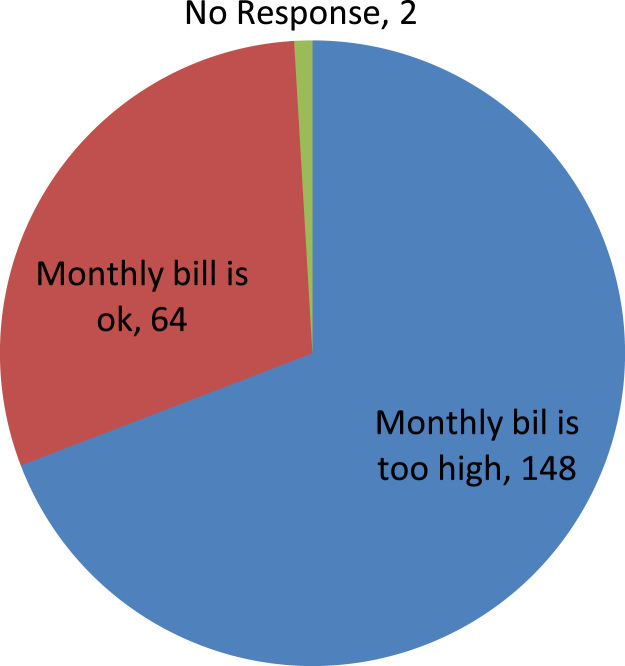
Respondents’ opinion on DISCO monthly charges.

**Fig. 10:**
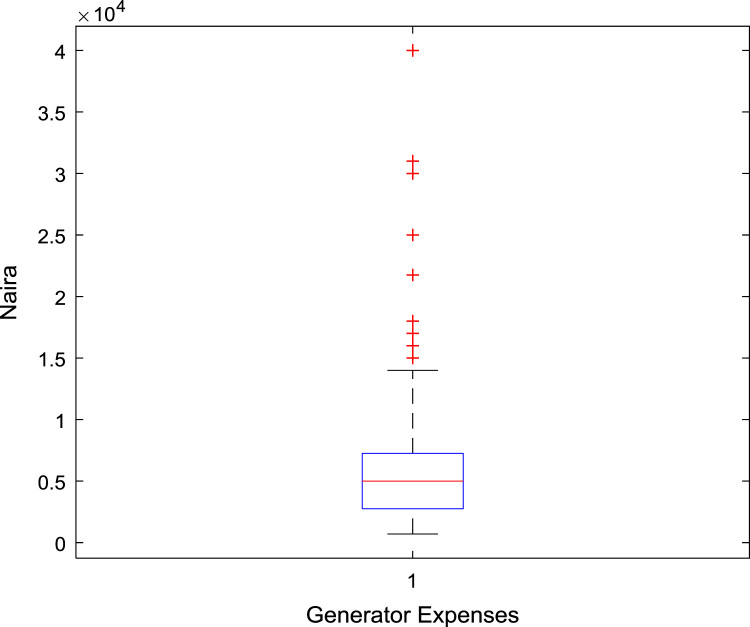
A box plot of the average monthly expenses on generator.

**Fig. 11:**
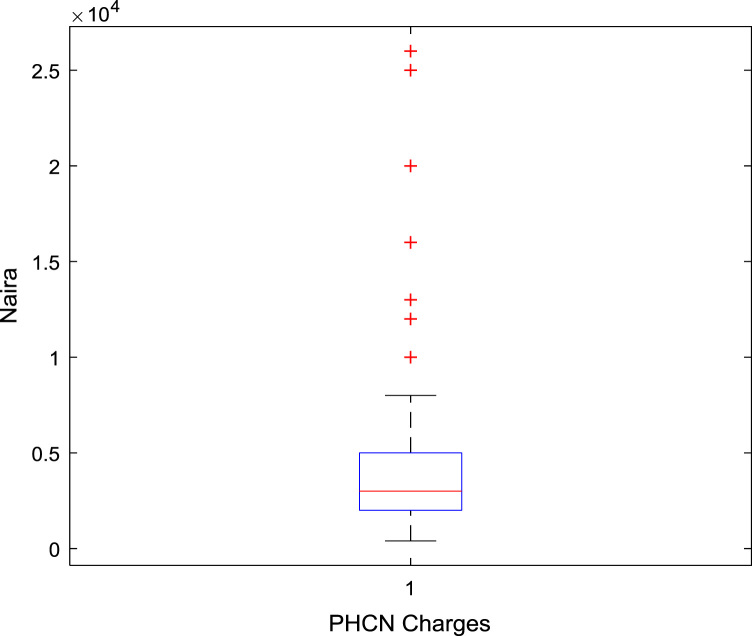
A box plot of the average DISCO Monthly Charges.

**Fig. 12:**
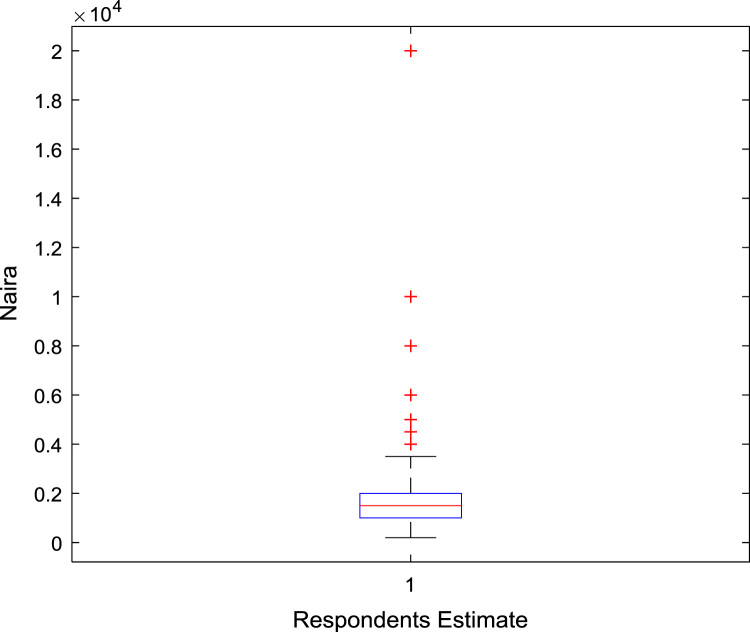
A box plot of respondents’ opinion of fair energy charge.

**Fig. 13:**
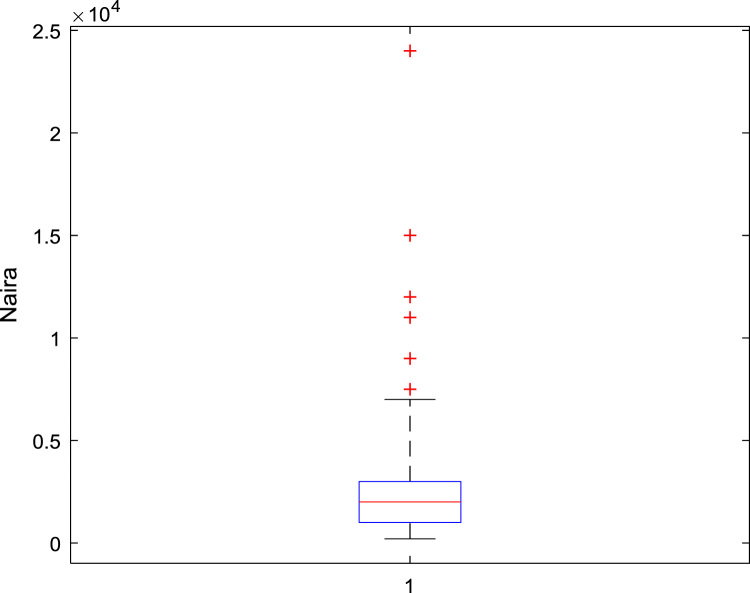
A box plot of the difference between DISCO Charges and Consumers’ Estimate.

**Fig. 14:**
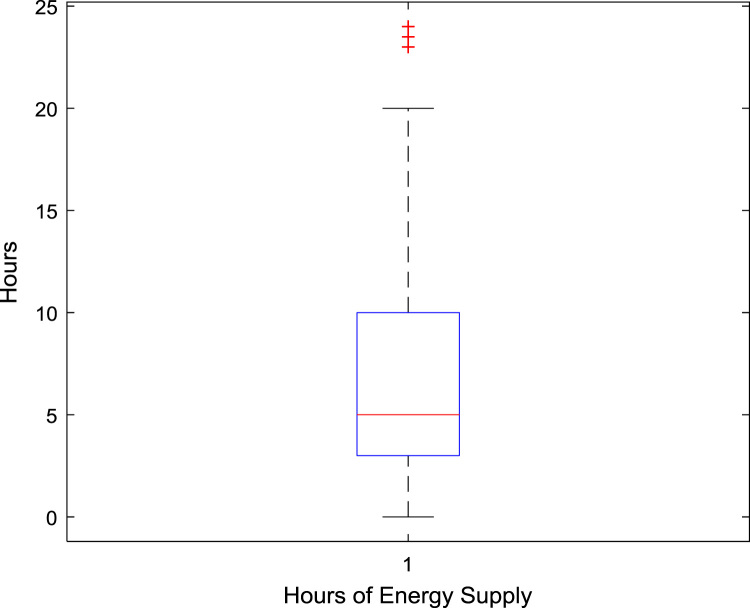
A box plot of the average number of hours of DISCO daily supply.

**Fig. 15:**
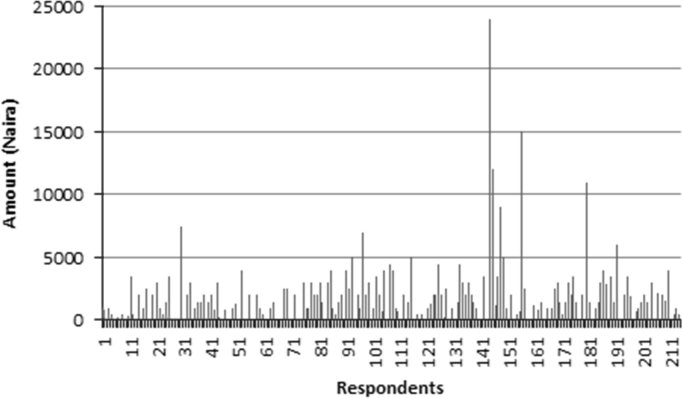
A chart showing the difference between DISCO Charges and Consumers’ Estimate.

**Fig. 16:**
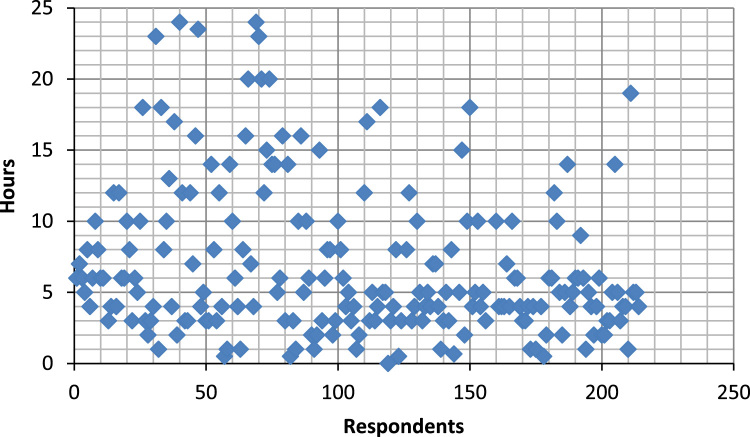
Scatter diagram of the average number of hours of DISCO daily supply.

**Fig. 17:**
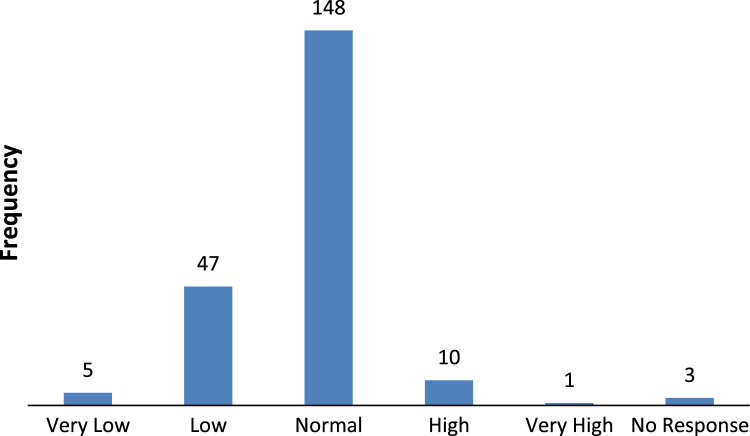
Respondents’ opinion of the quality of DISCO voltage supply.

**Fig. 18:**
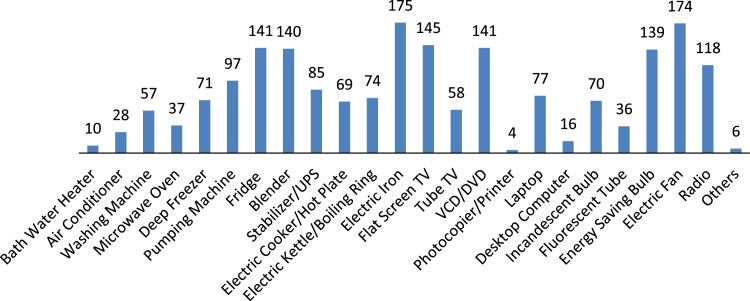
Electrical equipment distribution across sampled households.

**Fig. 19:**
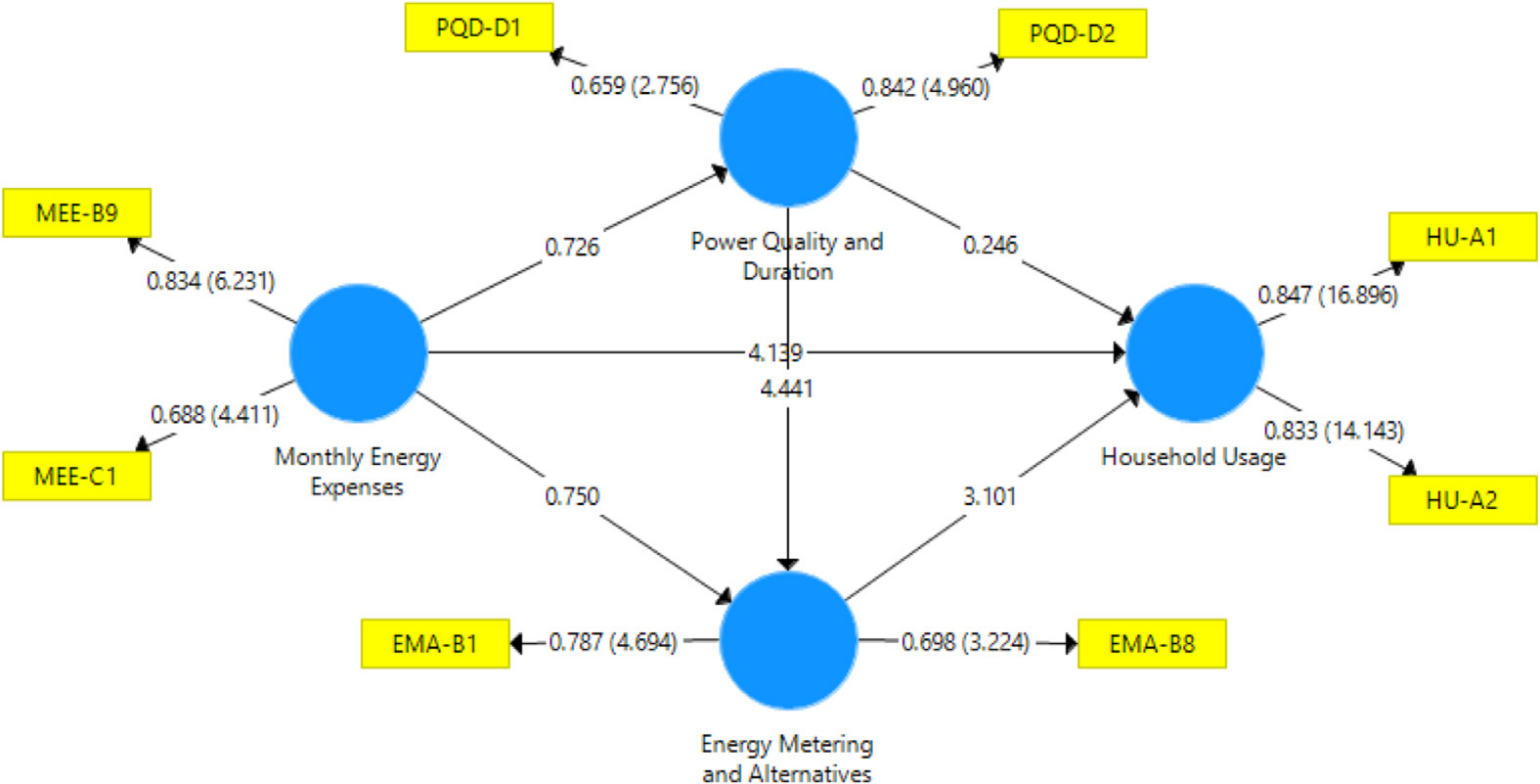
Statistical measurement model for the energy consumers’ data.

**Fig. 20:**
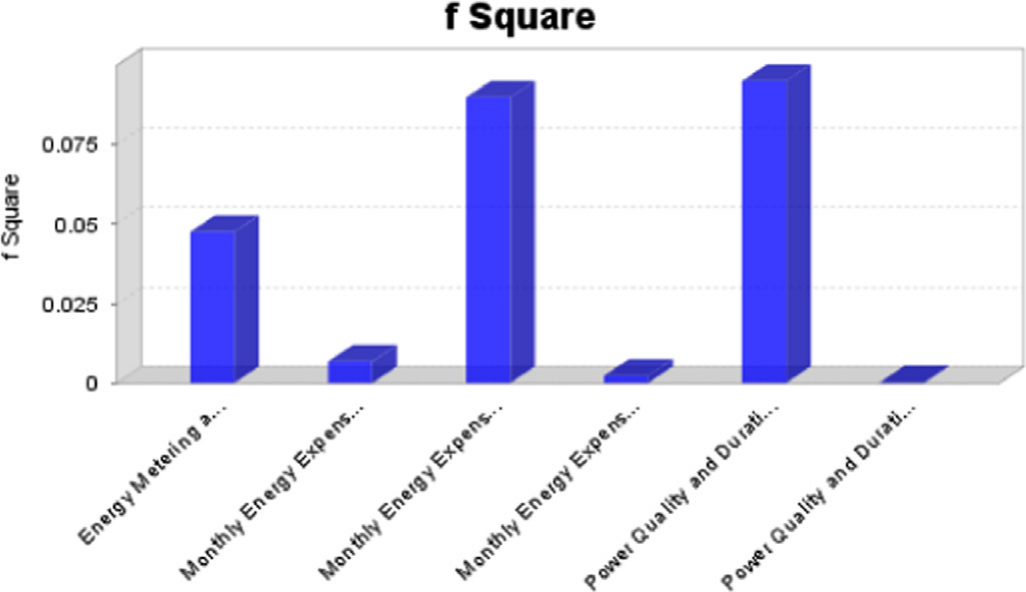
F square**:** effect size impact indicator (F square < 0.02 - No effect [17], [18]).

**Fig. 21:**
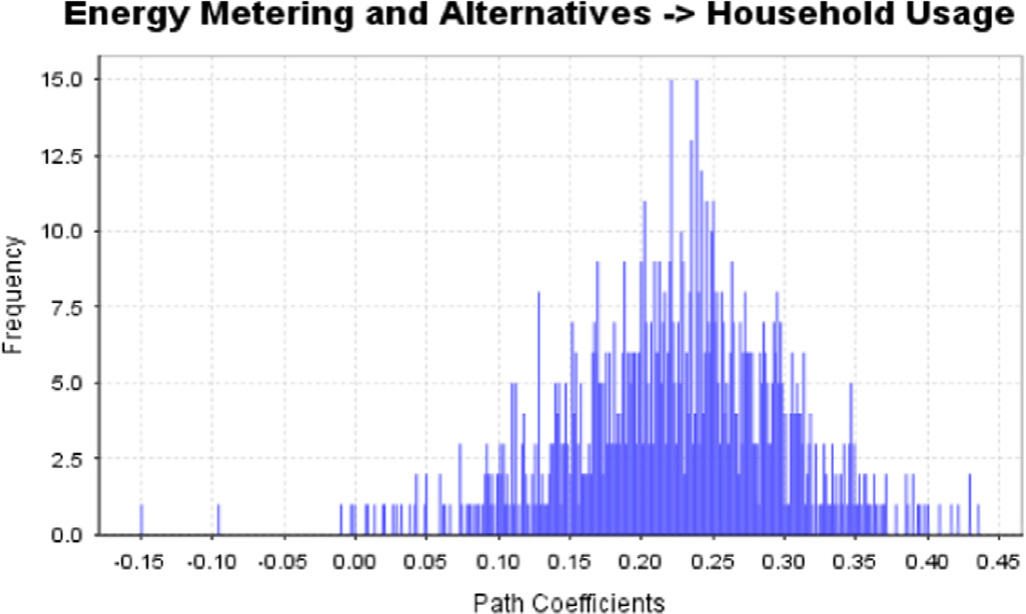
Path coefficient histogram for H1.

**Fig. 22:**
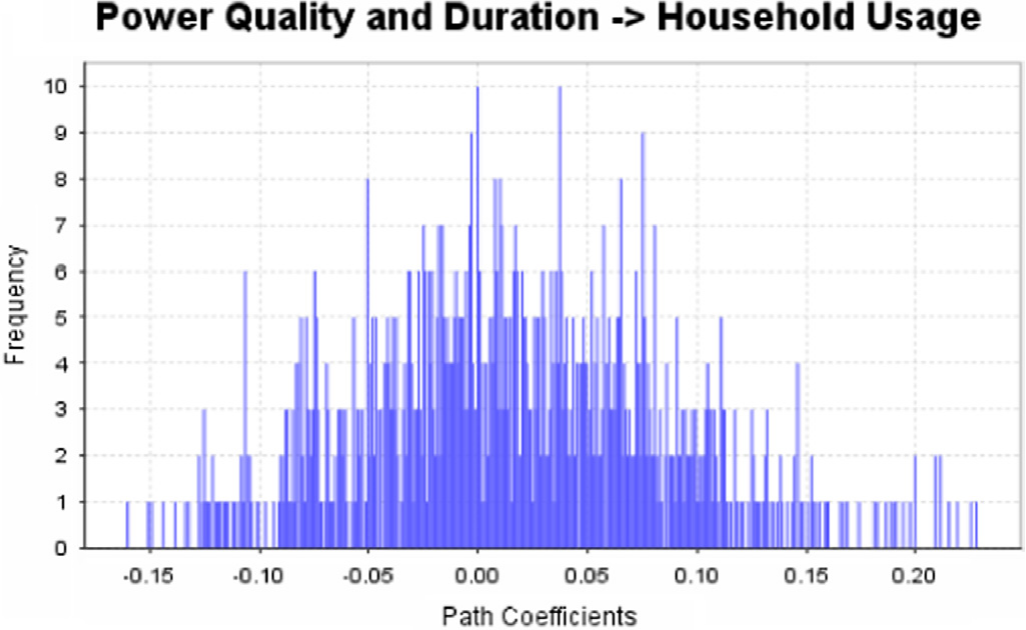
Path coefficient histogram for H2.

**Fig. 23:**
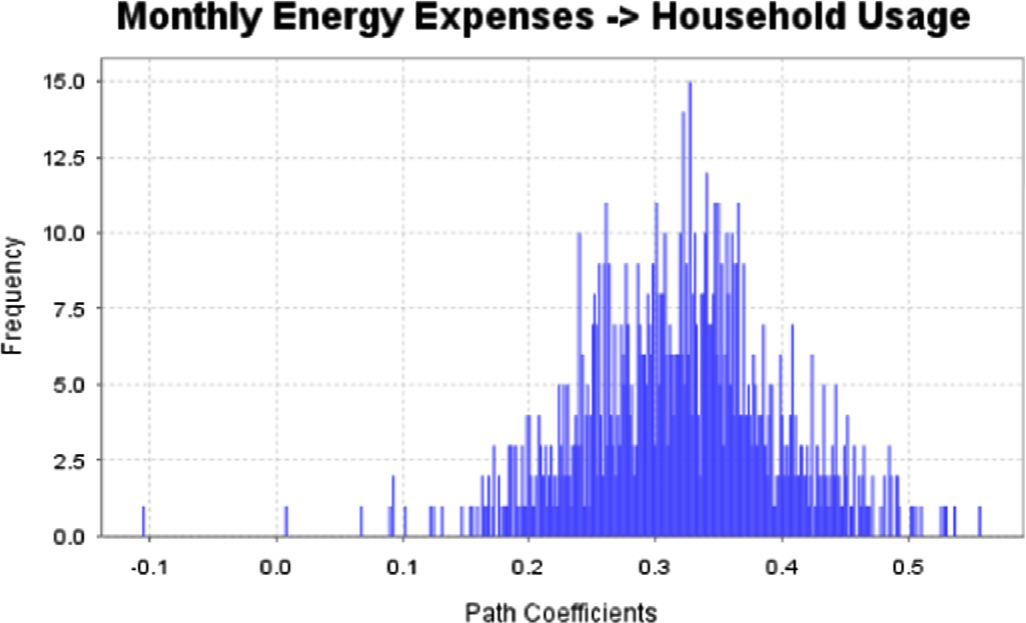
Path coefficient histogram for H3.

The data captures the different modes of electricity billing in Nigeria, and these are metered (prepaid and analog meters) and unmetered (estimated billing). Estimated billing often results in exorbitant charges termed “crazy bill” which is usually far above the actual energy consumption, and this is unfair to the customer [2]*.* The analog meter was the only alternative to estimated billing until in recent years with the advent of prepaid meters [3], [4]. Most of the analog meters were installed decades ago and are no longer accurate while some have been tampered with to slow their reading or to stop it completely from metering the energy consumed. NERC has mandated all DISCOs to install prepaid meters for all their customers but this is yet to be fully complied with, as DISCOs complain of lack of funds to procure the needed prepaid meters [5]. The data captures the proportion of the sampled residents that are on prepaid meters, it also reflects the opinion of the customers of their current monthly energy charges, it reflects the average hours of power supply by the DISCO to the residents due to insufficient power generation and load shedding [6], [7], [8], [9], [10], and also, it presents the profile of the commonly used electrical equipment, used within the community. According to [11], [12] the type of electrical equipment used and the behavioral energy usage trend determines a customer׳s monthly electricity bill. Fig. 1, Fig. 2 describe the types of accommodation sampled and the number of people per household, Fig. 3, Fig. 4, Fig. 5, Fig. 6, Fig. 7, Fig. 8, Fig. 9 captures the mode of energy billing, the view of energy consumers of DISCO charges, and the use of alternative energy sources to compensate inadequate public power supply [13]. Table 1 presents the descriptive statistics of the energy cost data while Fig. 10, Fig. 11, Fig. 12, Fig. 13, Fig. 14 present the boxplots of responses to questions on energy charges and average power supply duration. Fig. 15 shows the variation in Naira between DISCO׳s monthly charges and the expected fair usage estimate by each respondent. Fig. 16, Fig. 17, Fig. 18 detail the opinion of the respondent on the quality of the voltage supply and a summary of the electrical equipment used in the community. Various statistical analyses were performed on the dataset using methods similar to those found in [14], [15]. The statistical model of Fig. 19 was analyzed using Partial Least Squares approach to Structural Equation Modeling (SEM) [16]. Table 2 shows the Variance Inflation Factor (VIF) while Table 3 depicts the direct relationship of the hypothesis considered. Table 4 shows the establishment of the discriminant validity. The F square values are shown in Fig. 20 while the path coefficient histograms are illustrated in Fig. 21, Fig. 22, Fig. 23.

**Table 1:** Descriptive statistics of energy cost.

	**Monthly cost of running generator**	**Average DISCO monthly charges**	**Respondents cost estimate of actual consumption**	**Difference between DISCO charge and consumer׳s expectation**
**Mean**	6559.94	3675.85	1956.19	2446.17
**Sum**	1,023,350.00	779,280.00	410,800.00	364,480.00
**Min**	700.00	400.00	200.00	200.00
**Max**	40,000.00	26,000.00	20,000.00	24,000.00
**Range**	39,300.00	25,600.00	19,800.00	23,800.00
**Variance**	44,655,820.00	11,270,112.55	3,698,621.78	7,528,734.59
**Standard Deviation**	6682.50	3357.10	1923.18	2743.85
**Standard Error of Mean**	535.03	230.57	132.71	224.79
**Median**	5000.00	3000.00	1500.00	2000.00
**Mode**	5000.00	2000.00	1000.00	2000.00
**Count**	156	212	210	149

**Table 2:** Collinearity Statistics - Variance Inflation Factor (VIF).

	**Energy metering and alternatives**	**Household usage**	**Monthly energy expenses**	**Power quality and duration**
**Energy metering and alternatives**		1.100		
**Household usage**				
**Monthly energy expenses**	1.002	1.009		1.000
**Power quality and duration**	1.002	1.098		

VIF<5 - Acceptable (No strong indication of multicollinearity).

**Table 3:** Direct Relationships for Hypothesis testing.

**Hypothesis**	**Relationship**	**Std. beta**	**Std. error**	***t*-value**	***p* Values**	**95% CL LL**	**95% CL UL**
**H1**	Energy Metering and Alternatives ->Household Usage	0.222	0.071	3.101**	0.002	0.106	0.335
**H2**	Power Quality and Duration ->Household Usage	0.012	0.069	0.246	0.806	− 0.095	0.124
**H3**	Monthly Energy Expenses ->Household Usage	0.321	0.073	4.139**	0	0.199	0.446
**H4**	Monthly Energy Expenses ->Power Quality and Duration	− 0.052	0.073	0.726	0.468	− 0.173	0.061
**H5**	Power Quality and Duration ->Energy Metering and Alternatives	0.289	0.063	4.441**	0	0.184	0.382
**H6**	Monthly Energy Expenses ->Energy Metering and Alternatives	0.069	0.11	0.75	0.454	− 0.12	0.22

CL LL – Confidence Limit Lower Limit.

CL UL – Confidence Limit Upper Limit.

** *p* < 0.05 – Significant.

**Table 4:** Discriminant validity check using Fornell-Larcker Criterion.

	**Energy metering and alternatives**	**Household usage**	**Monthly energy expenses**	**Power quality and duration**
Energy metering and alternatives	0.789			
Household usage	0.236	0.86		
Monthly energy expenses	0.064	0.293	0.849	
Power quality and duration	0.29	0.065	− 0.048	0.766

## 2 Experimental design, materials and methods

These data were obtained by means of an investigative instrument captured by the questionnaire shown in the Appendix. 214 households were investigated and the obtained data fairly represent the various types of apartment available. The instrument is divided into seven parts labelled A to G. Part A was used to obtain the data regarding the type of apartment and the number of occupants. Part B covers methods of billing, metering information, and alternative back up generation used by consumers. Data on the average monthly payment was obtained in Part C, while Part D obtained the average daily duration and voltage level quality of electricity supply. In Part E, information relating to occupation and monthly income were gathered while Part F captured the electrical appliances used in each apartment. Lastly, the behavioural energy usage pattern is obtained from Part G. The collected data was processed, and various statistical analyses were carried out to determine the influence of power supply quality, the average duration of power supply per day, households’ monthly expense on energy, the use of energy meters and the availability of alternative energy sources on the anticipated households energy demand, using reflective constructs.
